# Yeast prions form infectious amyloid inclusion bodies in bacteria

**DOI:** 10.1186/1475-2859-11-89

**Published:** 2012-06-25

**Authors:** Alba Espargaró, Anna Villar-Piqué, Raimon Sabaté, Salvador Ventura

**Affiliations:** 1 Institut de Biotecnologia i de Biomedicina, Universitat Autònoma de Barcelona, E-08193, Bellaterra, Spain; 2 Departament de Bioquímica i Biologia Molecular, Facultat de Ciències, Universitat Autònoma de Barcelona, E-08193, Bellaterra, Spain; 3 Departament de Fisicoquímica, Facultat de Farmàcia, Universitat de Barcelona, Avda. Joan XXIII s/n, E-08028, Barcelona, Spain; 4 Institut de Nanociència i Nanotecnologia (IN2UB), Barcelona, Spain

**Keywords:** Protein aggregation, Inclusion bodies, Prions, Sup35-NM, Ure2p, Amyloid fibrils, *E. coli*

## Abstract

**Background:**

Prions were first identified as infectious proteins associated with fatal brain diseases in mammals. However, fungal prions behave as epigenetic regulators that can alter a range of cellular processes. These proteins propagate as self-perpetuating amyloid aggregates being an example of structural inheritance. The best-characterized examples are the Sup35 and Ure2 yeast proteins, corresponding to [*PSI+*] and [*URE3*] phenotypes, respectively.

**Results:**

Here we show that both the prion domain of Sup35 (Sup35-NM) and the Ure2 protein (Ure2p) form inclusion bodies (IBs) displaying amyloid-like properties when expressed in bacteria. These intracellular aggregates template the conformational change and promote the aggregation of homologous, but not heterologous, soluble prionogenic molecules. Moreover, in the case of Sup35-NM, purified IBs are able to induce different [*PSI+*] phenotypes in yeast, indicating that at least a fraction of the protein embedded in these deposits adopts an infectious prion fold.

**Conclusions:**

An important feature of prion inheritance is the existence of strains, which are phenotypic variants encoded by different conformations of the same polypeptide. We show here that the proportion of infected yeast cells displaying strong and weak [*PSI+*] phenotypes depends on the conditions under which the prionogenic aggregates are formed in *E. coli*, suggesting that bacterial systems might become useful tools to generate prion strain diversity.

## Background

Mammalian prions cause fatal neurodegenerative disorders, like Creutzfeldt–Jacob disease in humans, bovine spongiform encephalopathy and scrapie in sheep [1]. In yeast, several polypeptides can form prions that behave as dominant non-Mendelian cytoplasmic genetic elements. The best-characterized yeast prionogenic proteins are Sup35 and Ure2, which, in their aggregated state, form two cytosolic inheritable elements named *PSI+* and *URE3*, respectively. Whether this property is detrimental and prion formation constitutes a pathological yeast trait or it is, in contrast, associated to beneficial phenotypes is controversial [2]. The fact that in wild-type yeast, the *PSI+* or *URE3* prions were initially not found was interpreted in favour of the first possibility [3,4], but a recent study by the Lindquist’s group demonstrates that various yeast prions can be found in several isolates of wild type yeast [5], favouring thus the second possibility. Regardless of their cellular effects, both mammalian and fungal prion proteins are characterized by a high propensity to assemble into amyloid-like aggregates under physiological conditions both *in vitro* and in the cell [6]. Prions represent a particular subclass of amyloids for which the aggregation process becomes self-perpetuating *in vivo* and therefore infectious [7]. *In vitro*, the assembly of prions into amyloid aggregates displays a characteristic lag phase, which is abrogated in the presence of preformed fibres [8-10]. This seeded catalysis of the polymerization reaction underlies prion conformational replication and infectivity [6]. Reconstitution of *in vivo* infectivity from *in vitro* aggregates formed by recombinant purified prions has definitively proven the protein only hypothesis for prion formation and the connection between amyloid conformations and prion spreadable species [11,12]. Prion assemblies of the same protein might lead to phenotypically different transmissible states or strains [13]. It is suggested that this phenomenon results from a single protein being able to adopt multiple misfolded conformations, each one cor-responding to a specific strain.

The formation of inclusion bodies (IBs) in bacteria has long been regarded as an unspecific process depending on the establishment of hydrophobic contacts between partially or totally unfolded species after protein synthesis at the ribosome [14]. However, an increasing body of evidence indicates that bacterial IBs share a number of common structural features with the highly ordered and, in many cases, pathogenic amyloid fibrils [15-18]. So far, the conformational and functional characteristics of the IBs formed by prions in bacteria have been only explored in detail for the HET-s prion of the filamentous fungus *Podospora anserina*[19,20]. The HET-s prion functions in a genetically programmed cell-death phenomenon, which occurs when two fungal strains of different genotypes fuse [21]. For this particular prionogenic protein, the formation of IBs and amyloid fibrils seems to be a remarkably similar process as IBs display a highly ordered amyloid-like conformation at the molecular level [19,20], are able to seed the polymerization of amyloid-fibrils *in vitro*[19,20] and turn to be infectious *in vivo*[20]. This suggests that the aggregates formed by other prionogenic proteins in bacteria might exhibit equal properties. We show here that this is the case for the yeast prion domain of Sup35 (Sup35-NM) and the Ure2 protein (Ure2p).

## Results and discussion

### Ure2p and Sup35-NM form β-sheet enriched IBs

**Figure 1:**
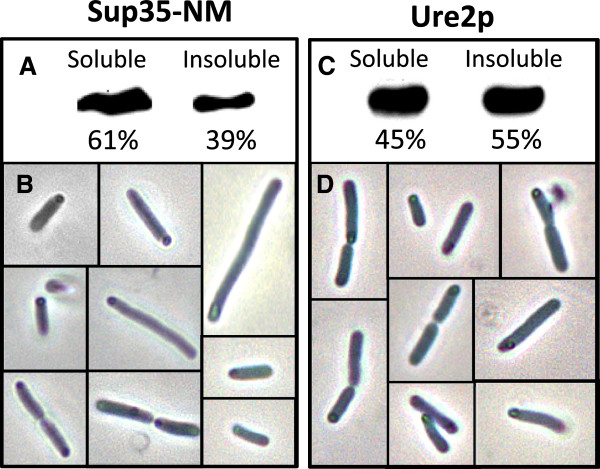
**Solubility properties of recombinant Sup35-NM (left panel) and Ure2p (right panel) proteins.** (**A** and **C**) Western blot of the soluble and insoluble fractions of cells expressing Sup35-NM and Ure2p at 37°C detected with an anti-histag antibody and quantified by densitometry using the Quantity-One software (Bio-Rad). (**B **and **D**) Localization of cytoplasmic IBs at the poles of cells expressing Sup35-NM and Ure2p proteins, as imaged by phase contrast microscopy.

We analyzed the cellular distribution of Ure2p and Sup35-NM proteins when expressed recombinantly in bacteria at 37°C. Western blotting and densitometry of the soluble and insoluble fractions indicate that about 50% of Ure2p and 40% of Sup35-NM recombinant proteins reside in the insoluble cellular fraction in these conditions (Figure 1A and C). Accordingly, bacterial cells expressing these polypeptides form birefringent IBs, located predo-minantly at the cell poles, as shown by phase contrast microscopy (Figure 1B and D).

**Figure 2:**
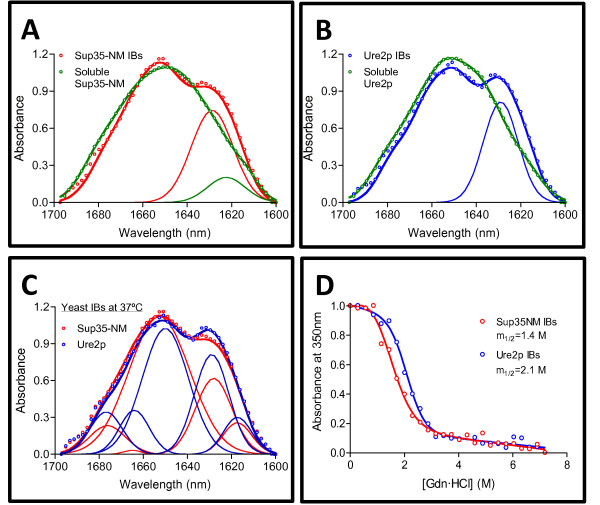
**Conformational properties of soluble and aggregated Sup35-NM and Ure2p proteins.** Secondary structure of Sup35-NM (**A**) and Ure2p (**B**) yeast proteins in their soluble forms and inside the IBs formed at 37°C as determined FT-IR spectroscopy in the amide I region of the spectrum. Empty circles, solid thick lines and solid thin line show the absorbance spectra, the sum of individual spectral components and the inter-molecular β-sheet band, respectively; note that whereas Sup35-NM and Ure2p IBs display the typical inter-molecular β-sheet band at 1625–1630 cm^-1^, this signal is low or absent in soluble species. (**C**) Comparative analysis of the secondary structure of Sup35-NM and Ure2p IBs. Empty circles, solid thick lines and solid thin lines show the absorbance spectra, the sum of individual spectral components and the deconvolved component bands, respectively. (**D**) Stability of yeast prionogenic IBs in front of Gdn·HCl denaturation at equilibrium monitored by changes in turbidity at 350 nm.

The aggregation of proteins into amyloid fibrils results in the formation of intermolecular β-sheets [22,23]. Fourier-transform infrared (FT-IR) spectroscopy allows addressing structural features of protein aggregates [24,25]. Specifically, the amide I region corresponding to the absorption of the carbonyl peptide bond group of the protein main chain is a sensitive marker of the protein secondary structure. To decipher the secondary structure in Sup35-NM and Ure2p IBs, we purified them from bacterial cell extracts and analyzed their FT-IR spectra (Figure 2A, B and C). Deconvolution of the absorbance spectrum in the amide I region for Sup35-NM and Ure2p IBs permitted to identify the individual secondary structure components and their relative contribution to the main absorbance signal. Both IBs exhibit FT-IR bands that can be assigned to the presence of intermolecular β-sheets (Table 1). These signals are absent or display a low intensity in the FT-IR of purified, initially soluble and monomeric, Sup35-NM and Ure2p species (Figure 2A and B). Therefore, as reported for other amyloid proteins [15,18,19,26], aggregation of Sup35-NM and Ure2p into IBs results in the formation of a supra-molecular structure in which at least part of the polypeptide chains adopt a disposition similar to this in amyloids. The IBs of the two yeast prionogenic proteins display, however, certain differences in secondary structure (Table 1 and Figure 2C); Ure2p IBs being slighted enriched in intermolecular β-sheet structure relative to Sup35-NM aggregates. The secondary structure content of Sup35-NM IBs closely resembles the one we observed for fibrils under agitation conditions [27]. In the case of Ure2p IBs, their secondary structure is more similar to that in fibrils formed under quiescent conditions [28]. In fact we have shown that, in contrast to Sup35-NM, the secondary structure content of Ure2p is strongly dependent on the aggregation conditions [27].

**Table 1:** **Secondary structure bands in the absorbance FT-IR spectra of purified *E. coli *Sup35-NM and Ure2p IBs**

**18°C**		**37°C**				
**Sup35-NM IBs**		**Sup35-NM IBs**		**Ure2p IBs**		
**Band (cm**^-1^**)**	**Area (%)**	**Band (cm**^-1^**)**	**Area (%)**	**Band (cm**^-1^**)**	**Area (%)**	**Structure**
1615	4	1617	7	1617	8	Tyrosine ring
1629	29	1628	21	1629	26	β-sheet (inter-molecular)
1652	51	1653	65	1650	45	loop/β-turn/bend/α-helix
1665	2	1676	6	1664	10	
1677	12	1682	1	1677	11	

The presence of regular secondary structure inside IBs implies the existence of cooperative interactions involving the main and side chains of the polypeptides embedded in these aggregates. To confirm this extent, we used chemical denaturation with guanidine hydrochloride (Gdn·HCl). We have shown before that this approach allows to approximate the conformational stability of intracellular aggregates [29]. Ure2p and Sup35-NM IBs denaturation was measured by monitoring the changes in absorbance at 350 nm in a Gdn·HCl range from 0 to 8 M. We calculated [Gdn·HCl]_1/2_ for IBs solubilization under equilibrium conditions (20 h incubation) to be 1.8 M and 2.1 M for Sup35-NM and Ure2p IBs, respectively (Figure 2D). These values are close to the one observed for HET-s PFD IBs (1.6 M) [19] and in agreement with their relative intermolecular β-sheet content. The cooperative denaturation transitions observed for both IBs support the presence of selective contacts in at least a fraction of the molecules deposited inside them.

### Amyloid properties of Sup35-NM and Ure2p IBs

**Figure 3:**
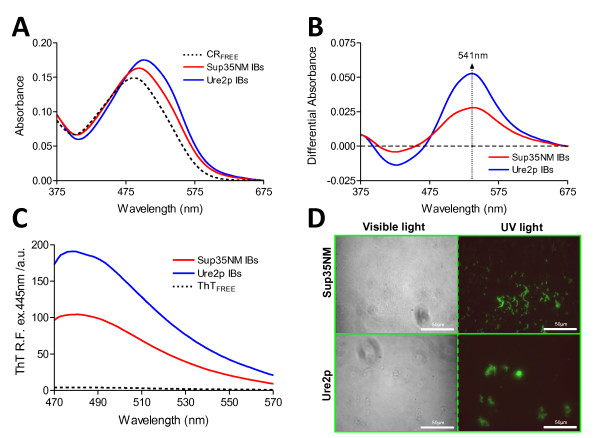
**Specific amyloid dyes staining of yeast prion IBs.** (**A**) CR spectral changes in the presence of each IB; displaying the characteristic red-shift in λ_max_ and intensity increase in CR spectra in the presence of IBs. (**B**) Difference absorbance spectra of CR in presence and absence of IBs showing the characteristic amyloid band at 541 nm for both yeast proteins. (**C**) Fluorescence emission spectrum of Th-T in the presence of each IB when excited at 445 nm; note the characteristic maximum at ~ 480 nm upon binding to amyloid structures. (**D**) Yeast prions IBs stained with Th-S and observed at 40x magnification by phase contrast and fluorescence microscopy displaying the green fluorescence characteristic of amyloid structures.

We used the amyloid-specific dyes Congo red (CR), thioflavin T (Th-T) and S (Th-S) to confirm that the detected β-sheet secondary structure in Sup35-NM and Ure2p IBs is organized into an amyloid-like suprastructure. The absorbance of CR increases and the spectrum maximum red-shifts to 510 nm in the presence of both IBs (Figure 3A). This spectral change corresponds to that observed in the presence of the fibrils formed *in vitro* by both proteins [27,30,31]. Moreover, the difference spectra of the dye in the presence and absence of IBs exhibit the characteristic amyloid band at 541 nm (Figure 3B).

Th-T fluorescence emission is enhanced in the presence of yeast prion amyloid fibrils [27,30,31]. The same behaviour is observed upon incubation of Th-T with yeast proteins IBs (Figure 3C). The Th-T fluorescence at the 480 nm spectral maximum increases 20- and 40-folds for Sup35-NM and Ure2p IBs, respectively. Furthermore, binding of Th-S to IBs was visualized by fluorescence microscopy (Figure 3D). For both IBs, areas rich in fibrous material were stained with Th-S to yield a bright green–yellow fluorescence against a dark background. Therefore, consistently with the secondary structure data and the existence of selective interactions, the dye binding results indicate that both IBs possess detectable amounts of amyloid structure.

### Sup35-NM and Ure2p IBs selectively seed amyloid formation

**Figure 4:**
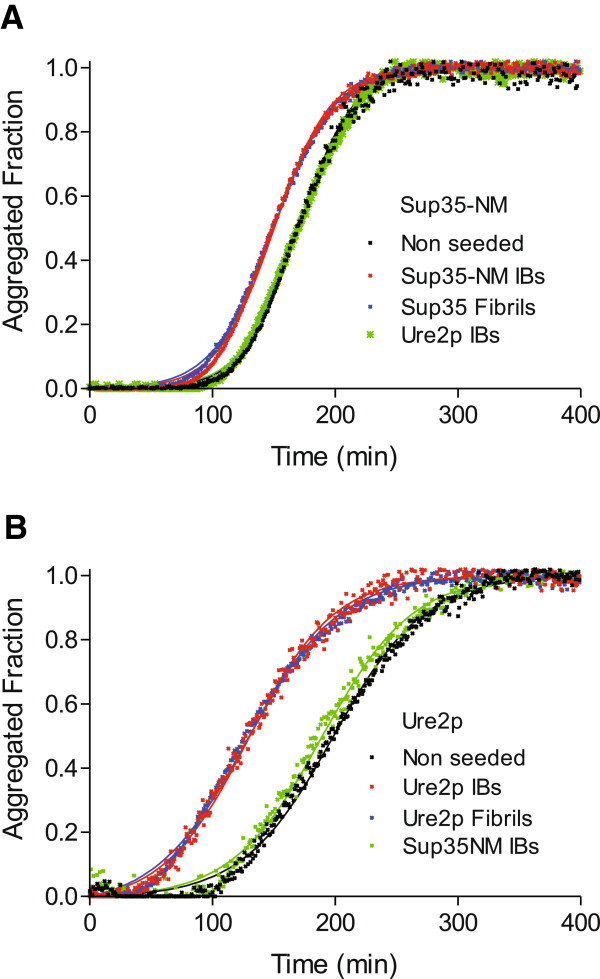
**Aggregation kinetics of Sup35-NM and Ure2p.** The aggregation reactions of 20 μM yeast prionogenic proteins were carried out under agitation at 37°C. 2 μM of *in vitro* formed fibrils (representing 10% of the final protein concentration) or IBs (at a final OD_350nm_ of 0.125) were used for seeding and cross-seeding assays. The fibrillar fraction of Sup35-NM (**A**) and Ure2p (**B**) is represented as a function of time. The formation of Sup35-NM and Ure2p amyloid fibrils are accelerated only in the presence of pre-aggregated homologous protein, either fibrils or IBs.

The kinetics of amyloid fibril formation usually results in a sigmoid curve that reflects a nucleation-dependent growth mechanism [29]. We have shown previously that the *in vitro* assembly of Sup35-NM and Ure2p fibrils follows this kinetic scheme [27]. The detected lag phase corresponds to the formation of the initial nuclei on which the polymerization or fibril growth would further spontaneously proceed. Seeded protein polymerization is a well-established mechanism for *in vivo* amyloid fibril formation and underlies prion propagation [32-34]. In Figure 4, it is shown, the effect of the presence of preformed amyloid Sup35-NM and Ure2p fibrils on the kinetics of fibril formation. In the presence of a 10% of preformed fibrils, the apparent nucleation constant (*k*_n_) increases by three- and five-fold for Sup35-NM and Ure2p, respectively (Table 2). As a result, the lag phase of the reaction is shortened by 22 min for Sup35-NM and by 62 min for Ure2p. As expected, no significant changes in the apparent elongation constants (*k*_e_) were detected since fibril seeds act preferentially at the nucleation stage.

**Table 2:** Kinetic parameters of Sup35-NM and Ure2p aggregation reactions

**Protein**	**Parameters**	**Non seeded**	**Sup35-NM Fibrils**	**Sup35-Nm IBs**	**Ure2p IBs**
Sup35-NM	*k*_n_ /10^6^*·*s^-1^	0.35	1.07	1.00	0.45
	*k*_e_ /M^-1^*·*s^-1^	37.54	36.08	36.67	36.07
	*c·k*_e_ /10^6^*·*s^-1^	750.83	721.50	733.33	721.33
	*t*_0_ /s	124.0	102.5	98.6	123.0
	*t*_1/2_ /s	169.9	150.1	149.6	171.0
	*t*_1_ /s	215.8	197.8	200.7	219.1
					
**Protein**	**Parameters**	**Non seeded**	**Ure2p Fibrils**	**Ure2p IBs**	**Sup35-IBs**
Ure2p	*k*_n_/10^6^*·*s^-1^	2.13	11.46	14.24	2.62
	*k*_e_ /M^-1^*·*s^-1^	22.33	23.40	21.49	22.53
	*c·k*_e_ /10^6^*·*s^-1^	446.67	468.00	429.83	450.50
	*t*_0_ /s	122.9	60.8	54.9	114.5
	*t*_1/2_ /s	199.9	131.2	129.5	190.3
	*t*_1_ /s	276.8	201.6	204.2	266.0

To test if the detected amyloid-like structures in Sup35-NM and Ure2p IBs were able to template the conformational conversion of their respective soluble species into amyloid fibrils, we performed aggregation experiments in the presence of preformed and purified IBs. The effect exerted by these aggregates on fibril formation kinetics is analogous to that promoted by the corresponding fibrillar states. Their presence do not affect *k*_e_ but increases *k*_n_ by three- and seven-fold for Sup35-NM and Ure2p reactions, respectively; shortening the respective lag phases in 26 min and 68 min (Figure 4). Interestingly enough, fibrils and IBs have quantitatively similar effects on the reaction constants for amyloid formation of yeast prionogenic proteins (Table 2).

In contrast to amorphous aggregation, amyloid formation is a specific process that can be seeded by homologous fibrils, but not by fibrils from unrelated polypeptides, even if they share a cross β-sheet conformation [35]. To test if this selectivity also applies in the case of IBs, we performed cross-seeding experiments, seeding the aggregation reaction of Sup35-NM with preformed Ure2p IBs and vice-versa. Importantly, the presence of heterologous prionogenic IBs does not affect the nucleation rates or lag times (Figure 4 and Table 2). This confirms that, as for fibrils, a specific molecular recognition between the soluble species and aggregated polypeptides underlies IBs-promoted fibril seeding.

**Figure 5:**
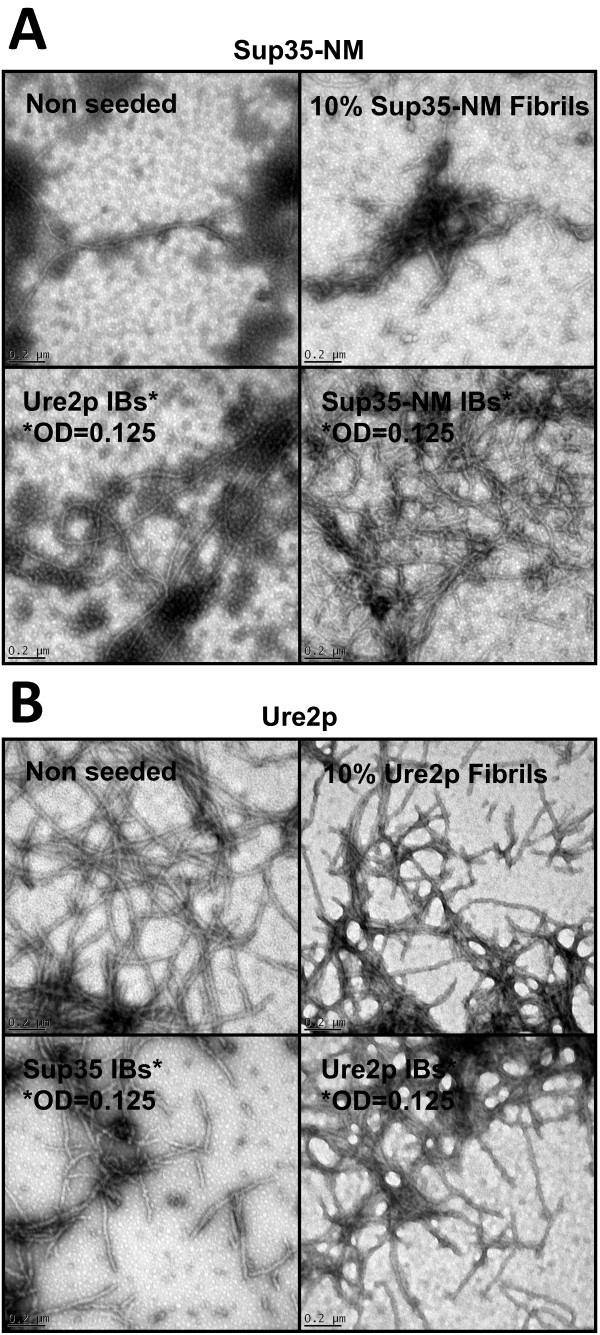
**Sup35-NM and Ure2p amyloid fibrils.** Morphology of Sup35-NM (**A**) and Ure2p (**B**) amyloid-like aggregates observed at the final time point of the aggregation kinetics. Fibrils in un-seeded, seeded and cross-seeded reactions were monitored by transmission electronic microscopy.

The morphology of the aggregates in seeded and non-seeded reactions was analyzed by transmission electronic microscopy (TEM) to make sure that the observed increase in aggregation rates results from a faster growth of amyloid material and not from a rapid formation of amorphous assemblies. As shown in Figure 5, regular fibrillar structures were observed in all cases. Interestingly, the morphology of the fibrils formed by seeding with fibrils and IBs of the same protein were similar. Overall, the data allow concluding that the selective intra- and inter-molecular contacts that characterize yeast prions fibrils are established as well by at least a fraction of the polypeptide chains embedded in the intracellular aggregates formed by these proteins in bacteria.

### Sup35-NM IBs are infectious

The Sup35 protein is an eukaryotic release factor, which is required for translation termination in yeast [36,37]. In contrast to *psi-* cells, where the Sup35 protein is soluble and functional, *PSI+* cells exhibit a nonsense suppressor phenotype due to reduced translation termination efficiency as consequence of the sequestration of native Sup35 into insoluble amyloid structures [38,39]. Both the cellular content of yeast *PSI+* cells and the amyloid fibrils formed *in vitro* by purified and soluble Sup35-NM are infectious and suffice to promote the transformation of the *psi-* phenotype into the *PSI+* if they enter the cell [40].

**Figure 6:**
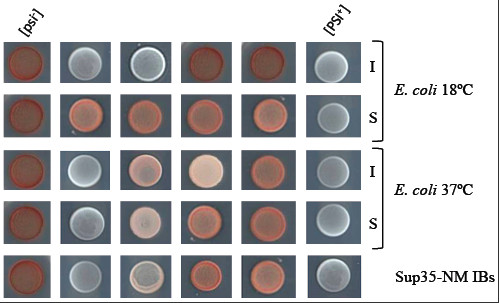
**Infectivity of Sup35-NM IBs.** Induction of different [*PSI+*] strains upon transformation of a [*psi*-] yeast strain with the soluble (S), insoluble (I) fractions of *E. coli* cells expressing Sup35-NM protein at 18 and 37°C or purified Sup35-NM IBs. After PEG transformation with the indicated material, yeast cells were recovered on SD-URA and randomly selected colonies were spotted onto ¼ YPD plates to identify [*PSI*^+^] converted colonies. [*psi*-] and [*PSI+*] columns correspond to the parental negative and positive control strains. Transformation with the bacterial material induced pink (weak) and to white (strong) [*PSI+*] phenotypes. Representative images of spots corresponding to distinct strains are shown for each transformed material (see Additional file 1: Table S1 for quantitative data).

The biophysical characterization of Sup35-NM and Ure2p aggregates suggests that these proteins might get access to prion conformations when expressed recombinantly in bacteria. As described above, in the case of Sup35-NM this property can be assessed by monitoring the conversion of *psi-* yeast cells into *PSI+* ones. To test this possibility, we fractionated bacterial cells expressing Sup35-NM. The resulting soluble and inso-luble fractions were used to transform spheroplasts of a *psi-* yeast strain as described in the Methods section. Bacterial cells expressing an insoluble variant of the spectrin SH3 domain (MAXF-SH3) [41] were processed in the same manner as a control, to make sure that phenotypic conversion is not caused by endogenous bacterial material or by the presence of a generic aggregation-prone protein in the transformation solution. A pESC-URA3 plasmid that allows selecting for the reduced fraction of transformed cells by uracil auxotrophy was added to each of the fractions. Upon spheroplast transformation, yeast cells were grown in uracil-deprived plates. Subsequently, they were streaked in ¼YPD plates. On these plates, *psi-* cells are of an intense red color whereas *PSI+* cells appear white or pink, depending if they convert to strong or weak *PSI+* strains, respectively [42]. No *PSI+* colonies were observed for transformations with any of the fractions of MAXF-SH3 expressing cells. In contrast, transformation with the soluble and insoluble fractions of Sup35-NM expressing bacteria resulted in a 1.7% and 3.5% of *PSI+* colonies, respectively (Figure 6 and Additional file 1: Table S 1). These results are reminiscent of those recently reported by Hochschild and co-workers using a fusion of a Sup35-NM^R2E2^ variant, containing extra copies of the critical oligopeptide repeat region and displaying an increased propensity to convert spontaneously into the prion form in yeast [43], to GFP. They convincingly demonstrated the formation of prionic variants of this protein fusion in bacteria [44]. In our study, we confirmed this behaviour using the wild type Sup35-NM domain without any mutation or fusion that might modify its intrinsic aggregation or conversion propensity [45].

**Figure 7:**
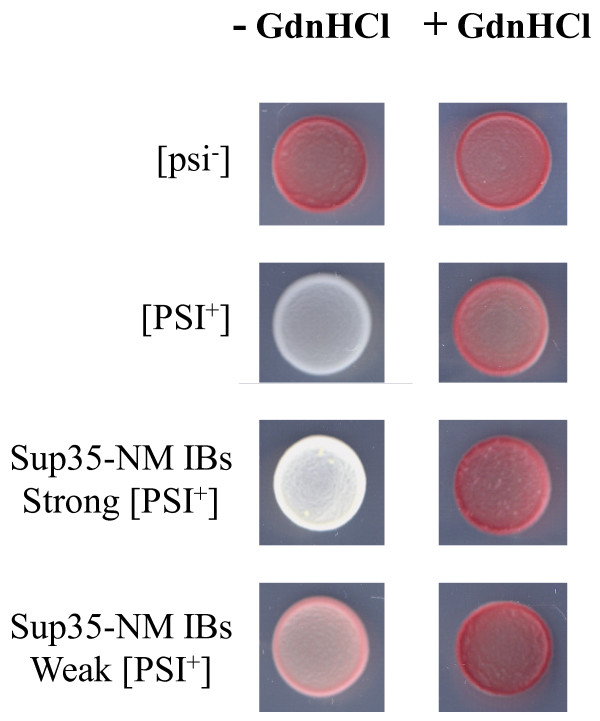
**Curing the Sup35-NM IBs induced [*PSI+*] phenotype.** Comparison of spots of control [*psi*-] and [*PSI+*] strains with cells displaying weak and strong [*PSI+*] phenotypes obtained by infection with Sup35-NM IBs. Cells were spotted on ¼ YPD before (left) and after (right) culture on a medium containing 3 mM Gdn·HCl.

An important difference between the results in both studies is that in the case of the Sup35-NM^R2E2^ -GFP fusion, the co-expression of the yeast New1 prionogenic protein in bacteria appeared as a requirement for prion formation. In contrast, our data argue that the natural bacterial protein machinery suffices to support the formation of prionic conformations, without a requirement for exogenous factors. This apparent discrepancy in the genetic background required for prion formation in bacteria might arise, among other reasons, from the fact that, in our hands, the Hochschild fractionation protocol causes precipitation and loss of most IBs. We thought that, according to their amyloid-like properties, the polypeptides embedded in these aggregates might contribute significantly to infectivity. To confirm this point, we purified Sup35-NM IBs from the insoluble fraction of cells cultured at 37°C and transformed them in [*psi-*] yeast spheroplasts. [*PSI+*] strain conversion occurred at a frequency of 5.6%. 65% of the transformed cells exhibited a weak pink [*PSI+*] phenotype and the rest where white (Figure 6 and Additional file 1: Table S 1). Both Sup35-NM IBs induced weak and strong [*PSI+*] phenotypes could be cured when the transformed yeast cells were transiently grown on a medium containing guanidine hydrochloride (Figure 7). Moreover, when cellular extracts of [*PSI+*] yeast cells resulting from IBs transformation were used to transform [*psi-*] spheroplasts, 40% of the resulting colonies converted to [*PSI+*]. These two features are characteristic of [*PSI+*] strains and support an infective prion nature for at least a fraction of the protein embedded in Sup35-NM IBs. Overall, independently of methodological differences, the data in the two studies converge to demonstrate that the bacterial cytosol supports the formation of infective amyloid-like structures.

### Temperature dependence of the infectious properties of Sup35-NM aggregates

**Figure 8:**
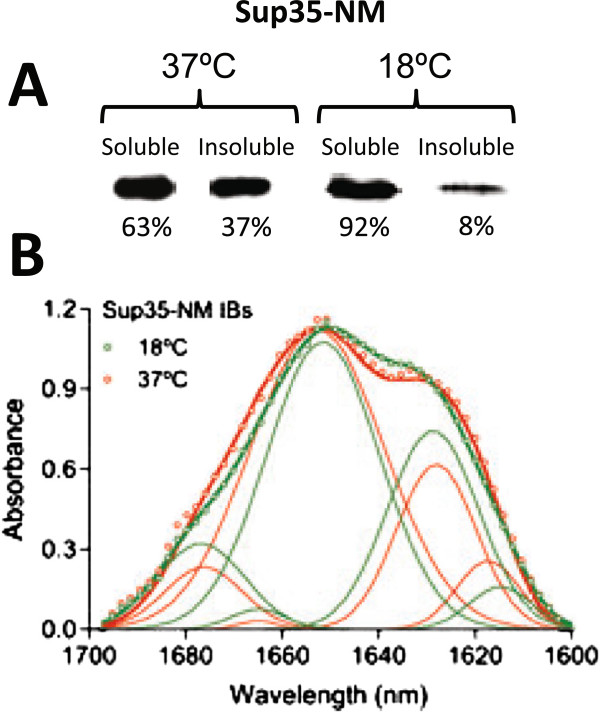
**Solubility and conformational properties of Sup35-NM as a function of the temperature.** (**A**) Western blot of the soluble and insoluble fractions of cells expressing Sup35-NM at 18 and 37°C detected with anti-histag antibody and quantified by Quantity One software. (**B**) Comparative analysis of the secondary structure of Sup35-NM IBs formed at 18°C and 37°C as determined FT-IR spectroscopy in the amide I region of the spectrum. Empty circles, solid thick lines and solid thin lines show the absorbance spectra, the sum of individual spectral components and the deconvolved component bands, respectively.

It is postulated that the existence of distinct amyloid conformations of Sup35-NM accounts for the different *PSI+* phenotypes that this prionogenic protein induces in yeast [40,46,47]. *In vitro*, the temperature at which the aggregation of prionogenic proteins occurs might influence the conformational properties of the resulting fibrils [27]. Accordingly, Weissman and co-workers demonstrated that Sup35-NM fibrils formed *in vitro* at different temperatures rendered different *PSI+* phenotypes when transformed into *psi-* cells. Fibrils formed at 4°C resulted in a majority of *PSI+* cells displaying a strong (white) phenotype whereas fibrils formed at 37°C rendered mostly weak (pink) strains [40]. This result is in agreement with our observation that most of the *PSI+* yeast strains obtained after transformation with the content of bacterial cells expressing Sup35-NM at 37°C displayed a weak phenotype. We wondered if, by analogy to fibrils, cultivation of Sup35-NM expressing cells at lower temperature would result in a significant increase of transformed cells displaying a strong phenotype. To this aim, Sup35-NM was expressed in bacterial cells grown at 18°C. First, we addressed if production at lower temperature modifies the distribution of recombinant Sup35-NM between the soluble and insoluble fractions. As it can be seen in Figure 8A, at 18°C the fraction of Sup35-NM protein residing in the insoluble fraction is reduced by about five-fold relative to that observed at 37°C, representing 8% of the total recombinant protein. This solubilizing effect of reduced temperature is well-documented for the expression of different model proteins [48]. Still, when the cellular fractions of these bacterial cells were used to transform *psi-* spheroplasts, the conversion efficiency into *PSI+* phenotypes was about five-fold higher for the insoluble fraction than for the soluble one (Figure 6 and Additional file 1: Table S 1), arguing that Sup35-NM aggregates are enriched in prion conformations relative to the corresponding soluble species. Interestingly enough, the reduction in the production temperature results in a significant increase in the proportion of white colonies (44%) among *PSI+* cells (Figure 6 and Additional file 1: Table S 1), relative to those observed at 37°C (25%). These data suggest that, in principle, one can modulate the infective properties of prionogenic proteins produced in bacteria by tuning the production conditions. In an effort to decipher the conformational determinants of the differential infective properties of 37 and 18°C insoluble fractions, we purified IBs from the low temperature insoluble fraction, analyzed their FT-IR in the amide I region of the spectra and compared it with the one of IBs obtained at 37°C (Figure 8B). The shapes of both spectra were fairly similar. This is in agreement with previous data in which we show that changes in the temperature of aggregation of Sup35-NM fibrils do not induce dramatic changes in their secondary structure content, as assessed by FT-IR [27]. Nevertheless, certain differences in the contribution of the spectral components to the main spectra could be detected. In particular, the ratio between the contribution of the band at 1628–1629 cm^-1^ and that at 1652–1653 cm^-1^ is higher in the IBs formed at 18°C (0.56) than in the IBs formed at 37°C (0.32), indicating a relative enrichment in intermolecular β-sheet in the 18°C aggregates [49] (Table 1). However, it is important to note that, despite the differences detected in IBs secondary structure content might contribute to the observed phenotypic differences between insoluble fractions, they might also be caused by more subtle conformational features to which FT-IR is blind, as shown for Sup35-NM amyloid fibrils [13].

## Conclusions

Prions are misfolded, self-propagating, infectious proteins. The bacterial IBs formed by HET-s PFD have been shown to display an amyloid fold and to be infective [19,20]. We show here that the IBs formed by the yeast Ure2p and Sup35-NM prionogenic proteins have an amyloid nature, while confirming the previous observation that bacteria supports the formation of Sup35-NM prion conformations. Moreover, we prove that a major fraction of the recombinant infective species is embedded in IBs. The formation of infectious prion folds in bacteria can be modulated by the expression conditions, as illustrated here using different growth temperatures. Since proteins accumulate in IBs at high levels and these biological particles are easily purified, it is suggested that they might become a convenient source to obtain prion particles exhibiting strain diversity. Besides, prion producing bacterial cells can potentially be used to develop screens for anti-prion drugs; an approach already validated in yeast models [50,51].

## Methods

### Protein expression and purification

Plasmids encoding Sup35-NM residues 1 to 254 (NM) C-terminally tagged with 7x-histidine and Ure2p N-terminally tagged with 6x-histidine have been described previously [13,52,53]. The histidine tag does not affect the biological activity of Sup35-NM and Ure2p in *Saccaromyces cerevisiae*[13,54]. The plasmids were transformed into BL21(DE3) pLysS *E. coli* cells. For protein expression, 10 mL overnight culture of transformed cells was used to inoculate 2 L of DYT medium, which was further incubated at 37°C and 250 rpm. At an OD_600nm_ of 0.5, protein expression was induced with 1 mM of isopropyl-1-thio-β-D-galactopyranoside (IPTG) at 37°C for 3 h and 14 h for soluble protein and IBs purification, respectively. The cultures were centrifuged at 8 000 x*g* for 10 min*,* then resuspended in 20 mL of deionized water, centrifuged at 15 000 x*g* for 10 minutes and the cell pellet was frozen at −80°C. For expression experiments at low temperature, cells were initially grown at 37°C until an OD_600nm_ of 0.4, transferred to 18°C for 20 min, induced with 1 mM IPTG and incubated for 14 h.

Ure2p and Sup35-NM proteins were purified from the soluble and insoluble cell fractions, respectively, essentially as previously described [27]. For lysis, cells were resuspended in 5 mL of deionized water and 45 mL of non-denaturing washing buffer (20 mM Tris·HCl at pH 8.0, 0.5 M NaCl) was further added. The cell suspension was placed under gentle agitation for 15 min. Finally, the samples were sonicated with a Branson Sonifier® ultrasonic cell disruptor for 3 min on ice. Soluble and insoluble fractions were separated after cell lysis by centrifugation at 15 000 x*g* for 30 minutes. When required, the insoluble fraction was resuspended in denaturing washing buffer. Affinity chromatography on FF-Histrap resin (Amersham, Uppsala, Sweden) under denaturing (20 mM Tris·HCl at pH 8.0, 0.5 M NaCl, 6 M Gdn·HCl, and 20 mM or 500 mM imidazole for washing and elution buffer, respectively) and non-denaturing conditions (20 mM Tris·HCl at pH 8.0, 0.5 M NaCl, and 20 mM or 500 mM imidazole for washing and elution buffer, respectively) was used for Sup35-NM and Ure2p purfication, respectively. Buffer was exchanged by gel filtration on Sephadex G-25 column (Amersham, Uppsala, Sweden) for native buffer (50 mM Tris·HCl and 150 mM NaCl at pH 7.4).

### Sup35-NM and Ure2p IBs purification

IBs were purified from induced cell extracts by detergent-based procedures as previously described [16]. Briefly, cells in a 10 mL culture were harvested by centrifugation at 12 000 x*g* (at 4°C) for 15 min and resuspended in 200 μL of lysis buffer (50 mM Tris·HCl pH 8.0, 1 mM EDTA, 100 mM NaCl), plus 30 μL of 100 mM protease inhibitor PMSF and 6 μL of a 10 mg/mL lysozyme solution. After 30 min of incubation at 37°C under gentle agitation, NP-40 was added at 1% (v/v) and the mixture was incubated at 4°C for 30 min. Then, 3 μL of DNase I and RNase from a 1 mg/mL stock (25μg/mL final concentration) and 3 μL of 1 M MgSO_4_ were added and the resulting mixture was further incubated at 37°C for 30 min. Protein aggregates were separated by centrifugation at 12 000 x*g* for 15 min at 4°C. Finally, IBs were washed once with the same buffer containing 0.5% Triton X-100 and once with sterile native buffer. After a final centrifugation at 12 000 x*g* for 15 min, pellets were stored at −20°C until analysis. The frozen pellets were reconstituted in native buffer. SDS-PAGE analysis revealed that in all cases the yeast proteins were the major polypeptidic components of the aggregates.

### Fibril formation: Aggregation kinetics and seeding assays

For aggregation reactions, 20 μM of soluble Sup35-NM and Ure2p in native buffer were placed under agitation (~750 rpm with micro-stir bars) at 25°C. Conversion of soluble species to aggregates was monitored by quantification of the relative Th-T fluorescence at 480 nm when exciting at 445 nm. In the seeding assay, a solution of yeast prion IBs (to a final OD_350nm_ = 0.125) or 2 μM of preformed fibrils was also added at the beginning of the reaction. Cross-seeding assays were performed in the same manner. Yeast prions aggregation process, as other related amyloid processes, may be modeled as an autocatalytic reaction using the equation *f* = (*ρ*{exp[(1 + *ρ*)*kt*-1})/{1 + ρ*exp[(1 + *ρ*)*kt*} under the boundary condition of *t* = 0 and *f* = 0, where *k* = *k*_e_a (when a is the protein concentration) and *ρ* represents the dimensionless value to describe the ratio of *k*_n_ to *k*. By non-linear regression of *f* against *t*, values of *ρ* and *k* can be easily obtained, and from them the rate constants, *k*_e_ (elongation constant) and *k*_n_ (nucleation constant). The extrapolation of the growth portion of the sigmoid curve to abscissa (*f* = 0), and to the highest ordinate value of the fitted plot, afforded two values of time (*t*_0_ and *t*_1_), which correspond to the lag time and to the time at which the aggregation was almost complete [9,27,55].

### Western blots

For Western blotting, bacterial cells were resuspended in lysis buffer and sonicated with a Branson Sonifier® ultrasonic cell disruptor for 3 min on ice. The cellular extract was centrifuged at 12 000 x*g* for 30 min. The soluble fraction was separated and pellet was resuspended exactly in the same volume of lysis buffer. To 50 μL of the soluble and resuspended insoluble fractions it was added 25 μL of loading buffer (180 mM Tris–HCl pH 7, 30% glycerol, 0.05% bromophenol blue, 9% sodium dodecyl sulfate (SDS) and 15% β-mercaptoethanol) and the mixture was heated at 95°C for 10 minutes. Insoluble and soluble fractions were resolved on 15% SDS–PAGE gels, transferred on to PVDF membranes, and recombinant proteins detected with a polyclonal anti-histag antibody. The membranes were developed with the ECL method [56]. The proportion of proteins in each fraction was determinated using Quantity-One analysis software (Bio-Rad, Hercules, CA, USA).

### Spheroplast preparation for transformation

#### Yeast cells culture

Yeast strains L1749 (*MATα ade1-14 ura3-52 leu2-3,112 trp1-289 his3-200*, [*psi*-], [*PIN*+]) and L1762 (*MATα adel-14 ura3-52 leu2-3,112 trp1-289 his3-200*, Strong [*PSI*+], [*PIN*+]) were kindly provided by Susan Liebman. Yeast strains were grown in solid YEPD medium for 48 h at 30°C; then a colony was inoculated in 10 mL liquid YEPD medium and incubated overnight at 30°C and agitation of 250 rpm. 5 mL of this culture were used to inoculate 50 mL of liquid YEPD at 30°C and 250 rpm. When an OD_600nm_ = 0.5 was reached, the culture was centrifuged at 1 500 x*g* and room temperature for 10 min. Cells were successively washed with 20 mL of sterile water and 1 M sorbitol, and centrifuged at 1 500 x*g* and room temperature for 5 min. Yeast cells were resuspended in SCE buffer (1 M sorbitol, 10 mM EDTA, 10 mM DTT, 100 mM sodium citrate at pH 5.8) and divided in 2 tubes.

#### Lyticase preparation

Lyticase from *Arthrobacter luteus* obtained as lyophilized powder, ≥200 units/mg solid (L4025: Sigma) was prepared at a final concentration of 10 000 units·mL^-1^ in phosphate buffer at pH 7.4 with 50% glycerol and kept at −80°C.

#### Spheroplast preparation

The first yeast cell tube was used to calculate the optimal spheroplast lyticase digestion time, according to the provider instructions. The second one was incubated with 10 μL of lyticase at 30°C until 85-90% of spheroplasts were reached. The spheroplasts solution was then centrifuged at 750 x*g* and room temperature for 10 min. The spheroplasts were gently resuspended and washed successively with 10 mL of 1 M sorbitol and STC buffer (1 M sorbitol, 10 mM CaCl_2_ and 10 mM Tris·HCl, pH 7.4), and centrifuged at 750 x*g* and room temperature for 10 min. Finally, the spheroplasts were gently resuspended in 100 μL of STC and immediately used.

#### Spheroplast transformation

25 μL of pelleted spheroplats resuspended in STC buffer were mixed with 3μL of sonicated soluble, insoluble fractions or IBs of Sup35-NM, URA3-marked plasmid (pRS316) (20 μg/mL) and salmon sperm DNA (100 μg/mL). Fusion was induced by addition of 9 volumes of PEG buffer (20% (w/v) PEG 8000, 10 mM CaCl_2_, 10 mM Tris·HCl at pH 7.5) for 30 min. Cells were centrifuged at 750 x*g* and room temperature for 10 min, and resuspended in SOS buffer (1 M sorbitol, 7 mM CaCl_2_, 0.25% yeast extract, 0.5% bacto-peptone), incubated at 30°C for 30 min and plated on synthetic medium lacking uracil overlaid with top agar (2.5% agar).

#### Analysis of prion phenotypes

After growth on synthetic medium lacking uracil (for >5 days), the efficiency of conversion from [*psi*-] to [*PSI*+] was tested by the following colour assay. Transformants were randomly selected and streaked onto ¼ YPD plates to enhance the colour phenotype. After 3 days the streaked colonies were classified as strong [*PSI*+] (white), weak [*PSI*+] (pink) and [*psi*-] (red) strains. The obtained conversion percentages result from the analysis of >500 colonies for each transformation assay.

#### Conversion from [PSI+] to [psi-] strains

Yeast strains with different phenotypes were grown in YEPD medium containing 3 mM of Gdn·HCl for 48 h at 30°C to cure the [*PSI*+] phenotype. The conversion from [*PSI*+] to [*psi*-] phenotype was assessed by spotting cells onto ¼ YPD plates.

### Secondary structure determination

ATR FT-IR spectroscopy analyses of Sup35-NM and Ure2p IBs were performed using a Bruker Tensor 27 FT-IR Spectrometer (Bruker Optics Inc) with a Golden Gate MKII ATR accessory. Each spectrum consists of 16 independent scans, measured at a spectral resolution of 1 cm^-1^ within the 1700–1500 cm^-1^ range. All spectral data were acquired and normalized using the OPUS MIR Tensor 27 software. FT-IR spectra were fitted to five overlapping Gaussian curves and the amplitude, centre, and bandwidth at half of the maximum amplitude and area of each Gaussian function were calculated using a nonlinear peak fitting program (PeakFit package, Systat Software, San Jose, CA).

### Chemical denaturation

For stability assays, purified IBs were prepared at OD_350nm_ = 1 in native buffer containing selected concentrations of guanidine hydrochloride (Gdn·HCl) ranging from 0 to 8 M. The reactions were allowed to reach equilibrium by incubating them for 20 h at room temperature. The fraction of soluble protein (*f*_*S*_) was calculated from the fitted values using equation: *f*_S_ = 1-((*y*_S_-*y*)/(*y*_S_-*y*_A_)), where *y*_S_ and *y*_A_ are the absorbance at 350 nm of the soluble and aggregated protein, respectively, and *y* is the absorbance of the protein solution as a function of the denaturant concentration.

The value m_1/2_ was calculated as the denaturant concentration at which *f*_S_ = 1/2. OD_350nm_ changes were monitored with a Cary400 Varian spectrophotometer.

### Binding of amyloid dyes to Sup35-NM and Ure2p IBs and amyloid fibrils

The interaction of 10 μM of Congo-Red (CR) with Sup35-NM and Ure2p IBs and fibrils was tested using a Cary100 UV/Vis spectrophotometer (Varian, Palo Alto, CA, USA) by recording the absorbance spectra from 375 nm to 675 nm using a matched pair of quartz cuvettes of 1 cm optical length placed in a thermostated cell holder at 25°C. In order to detect the typical amyloid band at ~541 nm, differential CR spectra in the presence and absence of protein were used.

The binding of 25 μM of Thioflavin-T (Th-T) to Sup35-NM and Ure2p was recorded using a Cary Eclipse spectrofluorometer (Varian, Palo Alto, CA, USA) with an excitation wavelength of 445 nm and emission range from 470 nm to 570 nm at 25°C in native buffer. For the staining assays with Thioflavin-S (Th-S), Sup35-NM and Ure2p IBs were incubated for 1 h in the presence of 125 μM of dye. After centrifugation (14 000 x*g* for 5 min), the precipitated fraction was placed on a microscope slide and sealed. Images of Sup35-NM and Ure2p IBs and fibrils bound to Th-S were obtained at 40-fold magnification under UV light or using phase contrast in a Leica fluorescence microscope (Leica DMRB, Heidelberg, Germany).

### Transmission electronic microscopy

Fibrils containing solutions were placed on carbon-coated copper grids, and left to stand for 5 min. The grids were washed with distilled water and stained with 2% (w/v) uranyl acetate for another two minutes before analysis using a HitachiH-7000 transmission electron microscope (Hitachi, Tokyo, Japan) operating at accelerating voltage of 75 kV.

## Competing interests

The authors declare that they have no competing interests.

## Authors' contributions

SV and RS supervised the project, designed the study and drafted the manuscript. AE carried out most of the experiments. AVP participated in the experimental work. All authors read and approved the final manuscript.

## Supplementary Material

Additional file 1 Table S1.Apparition frequencies of weak and strong [*PSI+*] phenotypes in the transformation of [*psi-*] yeast strain with the soluble, insoluble fractions of *E. coli* cells expressing Sup35-NM protein at 18°C and 37°C or purified Sup35-NM IBs. Click here for file
