# Supplementary material for: Yeast prions form infectious amyloid inclusion bodies in bacteria
Source: Microb Cell Fact. 2012 Jun 25;11:89. doi: 10.1186/1475-2859-11-89 (PMC3520751; doi:10.1186/1475-2859-11-89)

# Table1. Apparition frequencies of weak and strong [*PSI+*] phenotypes in the transformation of [*psi-*] yeast strain with the soluble, insoluble fractions of *E. coli* cells expressing Sup35-NM protein at 18º and 37 º C or purified Sup35-NM IBs


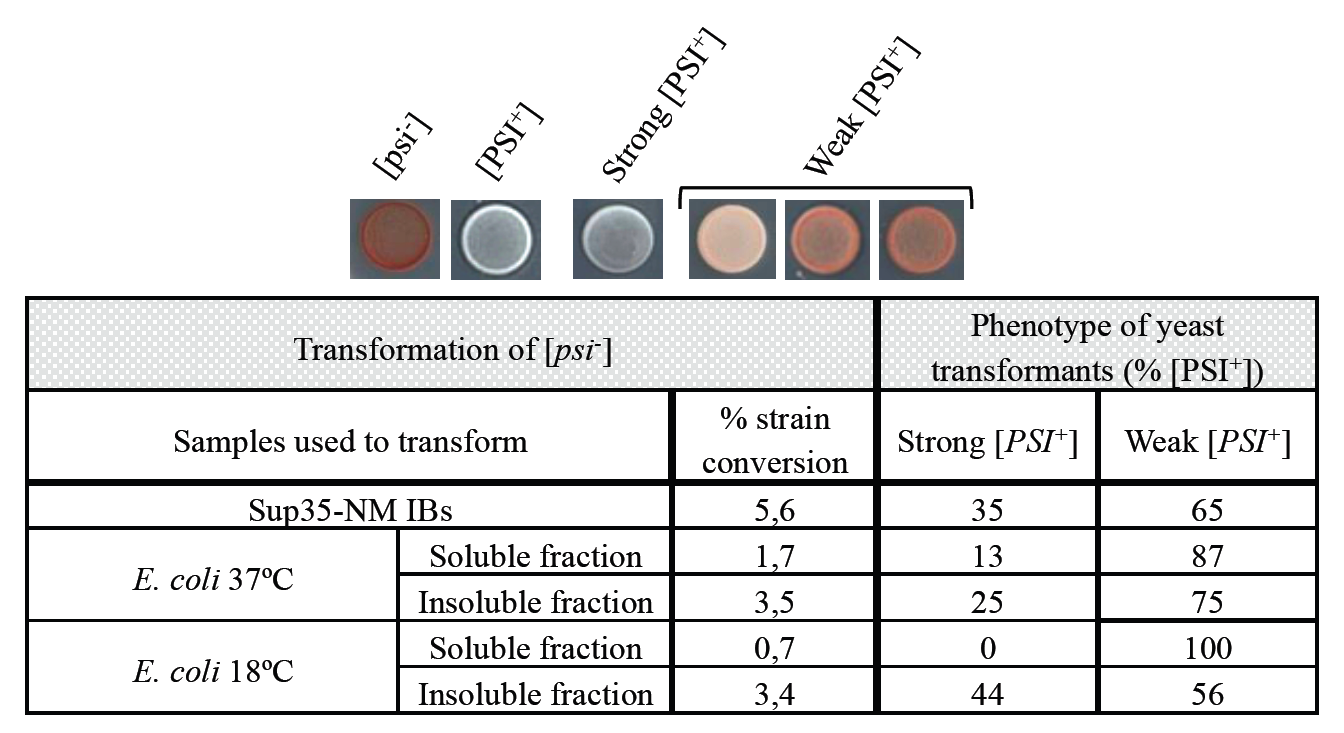

Supplement: Additional file 1 — Table S1.Apparition frequencies of weak and strong [PSI+] phenotypes in the transformation of [psi-] yeast strain with the soluble, insoluble fractions of E. coli cells expressing Sup35-NM protein at 18°C and 37°C or purified Sup35-NM IBs. [file 1475-2859-11-89-S1.doc]
